# Efficacy of HALP combined with T2-weighted MRI radiomics in predicting pathological complete response and overall survival in rectal cancer after neoadjuvant chemoradiotherapy: A retrospective single-center study in China

**DOI:** 10.1097/MD.0000000000050554

**Published:** 2026-09-11

**Authors:** Xiaolei Wan, Caiqiang Zhu, Dan Mei, Liang Liang, Sheng Zhang, Xiaolin Ge, Xinchen Sun, Zhengqi Li, Xiaoke Di, Ning Xue, Xiaojiao Chen

**Affiliations:** a Department of Oncology, Jurong Hospital Affiliated to Jiangsu University, Zhenjiang, Jiangsu Province, China; b Department of Clinical Research Coordination, Nanjing Yuzhong Medical Consulting Co., Ltd., Nanjing, Jiangsu Province, China; c Department of Radiotherapy Oncology, The First Affiliated Hospital of Nanjing Medical University, Nanjing, Jiangsu Province, China.

**Keywords:** HALP, neoadjuvant chemoradiotherapy, overall survival, pathological complete response, radiomics, rectal cancer

## Abstract

This study aimed to develop and validate an integrated model combining pretreatment hemoglobin, albumin, lymphocyte, platelet (HALP) score and T2-weighted magnetic resonance imaging radiomics for predicting pathological complete response (pCR) and overall survival in patients with locally advanced rectal cancer (LARC) undergoing neoadjuvant chemoradiotherapy. A total of 137 consecutive LARC patients from January 2019 to January 2022 were retrospectively included. Pretreatment 3.0T T2-weighted high-resolution magnetic resonance imaging scans were performed. Four stable features were selected using least absolute shrinkage and selection operator regression. The HALP score was calculated as (hemoglobin × albumin × lymphocytes)/platelets, with an optimal cutoff of 48.4 determined by X-tile analysis. Three predictive models were constructed: a radiomics-only model (RAD), a HALP-only model (HALP), and a combined model (RAD + HALP) using a random forest classifier. Model performance was assessed using the area under the curve (AUC), sensitivity, specificity, calibration curves, and decision curve analysis. The pCR rate was 13.1% (18/137). For pCR prediction, the combined model achieved an AUC of 0.808 (95% confidence interval: 0.73–0.88), compared to 0.788 for the RAD model. The HALP score alone did not show predictive capability (*P* = .59). For overall survival prediction, the combined model significantly outperformed the individual models, with an AUC of 0.957 (sensitivity: 0.945, specificity: 1.0; DeLong test, *P* = .002), the AUC was corrected to 0.852 (sensitivity: 0.788, specificity: 0.889; *P* < .001). Multivariate Cox analysis identified HALP as an independent prognostic factor (hazard ratio: 5.83, 95% confidence interval: 2.23–15.2). The combined RAD + HALP model demonstrated excellent and internally validated performance in predicting both short-term treatment response and long-term survival in LARC patients undergoing neoadjuvant chemoradiotherapy patients. Prospective multicenter studies are warranted prior to clinical application.

## 1. Introduction

Rectal cancer ranks as the 3rd most common gastrointestinal malignancy worldwide, with a rising incidence among individuals under 50 years of age.^[1,2]^ Locally advanced rectal cancer (LARC) is typically managed with neoadjuvant chemoradiotherapy (NCRT) followed by total mesorectal excision (TME); however, only 10% to 30% of patients achieve a pathological complete response (pCR).^[3,4]^ Accurate pretreatment prediction of pCR and long-term survival could help inform personalized treatment strategies, such as watch-and-wait approaches or therapy intensification.

In recent years, advances in medical imaging have enabled the extraction of high-dimensional features from tomographic images through high-throughput computing, converting visual data into quantifiable information – a process known as radiomics.^[5,6]^ Key steps in radiomics include image acquisition, segmentation, feature extraction, model development, and validation. Due to its high accuracy and practicality, radiomics has been widely investigated, and its potential in diagnosing and predicting outcomes across multiple malignancies is well recognized.^[7,8]^ Its utility in rectal cancer has also been validated.^[9]^ For instance, magnetic resonance imaging (MRI)-based radiomics models have been used to predict tumor response and pCR following chemoradiotherapy, potentially aiding in the selection of candidates for sphincter-preserving surgery.^[5,10,11]^ Recent multicenter MRI radiomics studies have reported area under the curve (AUC) values between 0.76 and 0.82 for pCR prediction^[12,13]^; however, most models still lack external validation or integration with systemic biomarkers.

Systemic inflammation and nutritional status significantly influence cancer outcomes. The hemoglobin, albumin, lymphocytes, platelets (HALP) score serves as a convenient composite indicator reflecting both inflammatory and nutritional status and has demonstrated prognostic value in gastric, esophageal, and colorectal cancers.^[14–16]^ Emerging evidence indicates that inflammatory pathways involving interleukin-11/ T helper17 and cluster of differentiation 40 signaling critically modulate the tumor microenvironment and host immune competence.^[17–19]^ Furthermore, programmed cell death protein 1-mediated immunosuppression has been shown to correlate with peripheral lymphocyte exhaustion, providing a mechanistic link between low lymphocyte counts (a component of HALP) and poor treatment response.^[20]^However, no study to date has integrated HALP with MRI radiomics to predict both short-term treatment response and long-term survival in LARC. We hypothesize that the combined model integrating tumor heterogeneity (via T2-weighted [T2W] radiomics) and host systemic status (via HALP) will outperform single-modality predictors in predicting pCR and long-term survival. The present study aims to develop and internally validate such a combined model in a Chinese LARC cohort undergoing NCRT.

## 2. Materials and methods

### 2.1. Patient selection and study design

A total of 187 patients with rectal cancer diagnosed and treated at our institution between January 2019 and January 2022 were retrospectively reviewed. The cohort included 130 males and 57 females. Inclusion criteria were as follows: biopsy-confirmed rectal adenocarcinoma with MRI-defined locally advanced disease (cT3–4 or node-positive, nonmetastatic); completion of NCRT followed by radical TME, with no prior antitumor therapy; availability of baseline MRI before NCRT; Zubrod/ECOG/WHO performance status of 0 to 2; and absence of active infection or autoimmune disease. Exclusion criteria included: treatment discontinuation; missing preoperative MRI or surgical data; lack of high-resolution T2W imaging; nonsurgical management; postoperative pathology showing mucinous adenocarcinoma or significant mucin on baseline MRI; and MRI performed on a 1.5T scanner.

**Figure 1.**
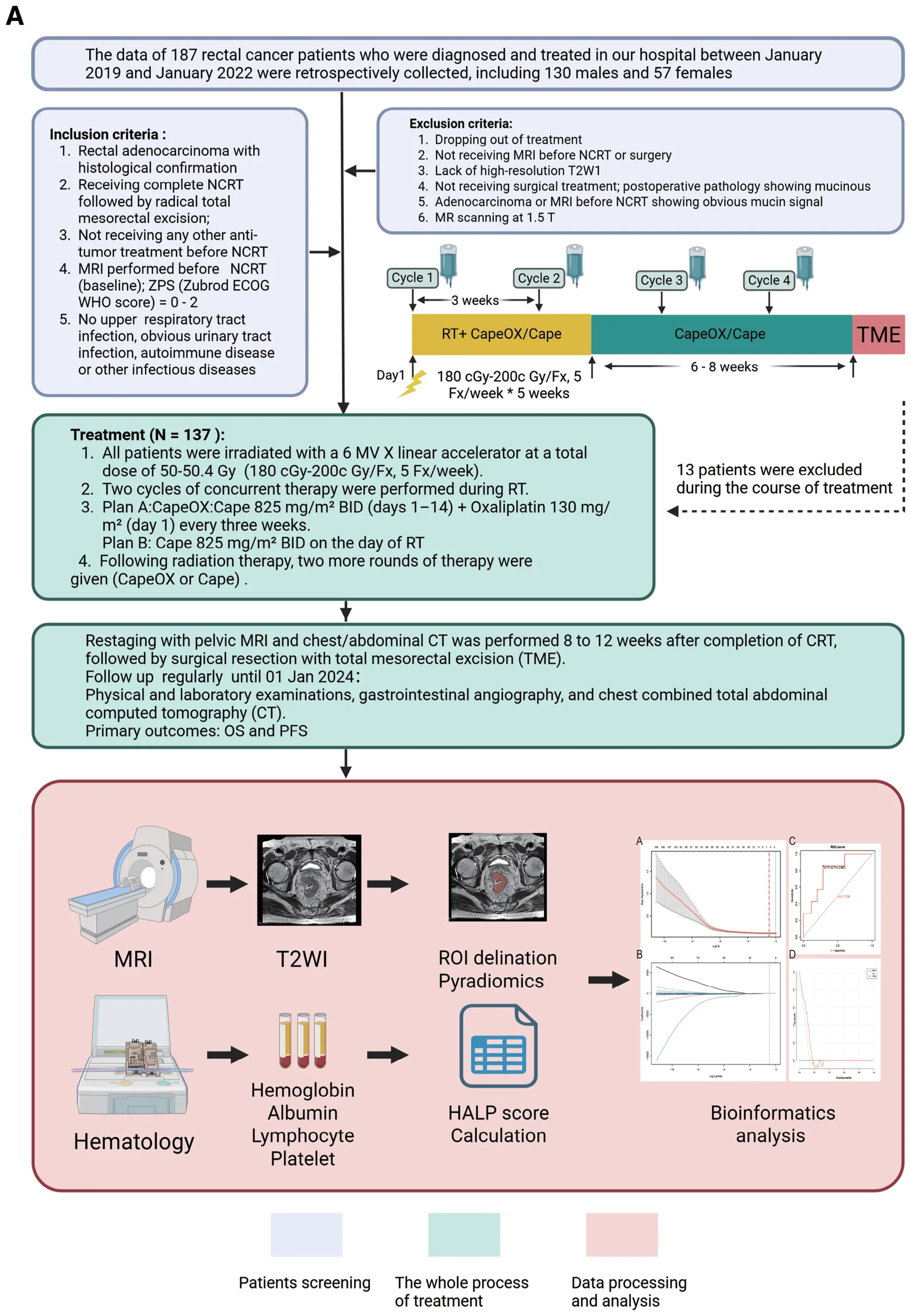
CONSORT flow chart of patient inclusion and radiomics analysis pipeline. CONSORT = consolidated standards of reporting trials. Created using the BioRender app (BioRender, Di X, 2026, https://BioRender.com/i30u824).

Reasons for exclusion: Of the 187 initially enrolled patients, 37 were excluded – 22 due to incomplete follow-up information, 11 due to distant metastasis detected during staging, and 4 lost to follow-up. Of the remaining 150 patients, an additional 13 were excluded: 10 due to treatment-related complications (grade 3–4 toxicity) and 3 who failed to complete treatment for personal reasons. Ultimately, 137 patients were enrolled (Fig. 1).

### 2.2. Treatment protocol and follow-up

All patients underwent intensity-modulated radiotherapy using a 6 MV X-ray linear accelerator, delivered in 1.8 to 2.0 Gy fractions 5 times/wk, to a total dose of 50 to 55 Gy. Concurrent chemotherapy consisted of oxaliplatin (50 mg/m^2^ weekly) plus capecitabine (625 mg/m^2^ orally twice daily on weekdays), or capecitabine alone, followed by radical TME performed 4 to 8 weeks after NCRT completion. Patients were followed regularly. pCR was defined as the absence of viable tumor cells in the primary tumor (ypT0) and regional lymph nodes (ypN0) after surgery, with overall survival (OS) defined as the time from NCRT initiation to death from any cause. Follow-up was conducted by telephone every 3 months for the 1st 2 years, every 6 months for the next 2 years, and annually thereafter until recurrence or death. Recurrence and metastasis were confirmed by computed tomography, MRI, positron emission tomography/computed tomography, or reoperation pathology. The final follow-up date was January 1, 2024. This study was approved by the Jurong People’s Hospital Ethics Committee (no. JRH-IEC-2024043), and written informed consent was obtained from all participants. The primary study endpoint was pCR prediction; secondary endpoints included OS.

### 2.3. MRI acquisition

MRI was performed using a 3.0T scanner (GE Signa Horizon) with an 8-channel phased-array coil. High-resolution axial T2W imaging parameters were: repetition time 3500 ms, echo time 110 ms, slice thickness 4 mm, gap 0.4 mm, matrix 288 × 288.

### 2.4. Image segmentation and analysis

Digital Imaging and Communications in Medicine images from pretreatment MRI were retrieved, and the high-resolution axial or axial oblique T2W sequence with a small field of view was prioritized for segmentation. If unavailable, the T2W sequence that best visualized the tumor was used. A radiation oncologist with 5 years of experience in rectal cancer manually segmented the entire rectal tumor and mesorectal compartment using 3D Slicer (version 4.0; www.slicer.org), blinded to pathological outcomes. All segmentations were verified by a senior radiation oncologist with 10 years of experience, with disagreements resolved by consensus. Interobserver intraclass correlation coefficients for manual segmentation between 2 radiologists ranged from 0.82 to 0.91 (Table S1, Supplemental Digital Content 1).

### 2.5. Radiomics feature extraction and selection

The radiomics workflow included the following steps: First, segmentation: whole-tumor regions of interest were manually delineated using ITK-SNAP (version 3.8) by a radiation oncologist with 5 years of experience, blinded to pathological outcomes. All segmentations were verified by a senior radiation oncologist with 10 years of experience, with disagreements resolved by consensus. Interobserver reproducibility: to assess segmentation stability, 30 randomly selected cases were independently segmented by 2 radiologists; intraclass correlation coefficients ranged from 0.82 to 0.91 (Table S1, Supplemental Digital Content 1). Second, preprocessing: *Z*-score normalization and resampling to 1 × 1 × 3 mm^3^ were applied. Third, feature extraction: a total of 14,796 radiomic features were extracted using PyRadiomics (v3.0.1), including 1st-order, shape, gray-level co-occurrence matrix, gray-level run-length matrix, gray-level size zone matrix, and Laplacian of Gaussian features. Fourth, feature selection: least absolute shrinkage and selection operator regression with 10-fold cross-validation (α_min_ = 0.032) was applied to the full dataset, retaining 4 stable features: low gray level emphasis, short run low gray level emphasis, small area low gray level emphasis, and low gray level run emphasis. Limitations: test–retest reproducibility and cross-scanner stability were not assessed due to the retrospective single-center design; this limitation is acknowledged in the Section 4.

### 2.6. Hematological parameter: HALP score

Baseline clinical and pathological data were collected within 1 week before NCRT, including gender, age, depth of invasion, tumor size, and differentiation grade. Hematological parameters – leukocyte, neutrophil, lymphocyte, and platelet counts, as well as hemoglobin, albumin, and globulin levels – were measured. The HALP score was calculated as: hemoglobin (g/L) × albumin (g/L) × lymphocyte count (/L)/platelet count (/L).^[21]^

### 2.7. Statistical analysis

Categorical variables are presented as frequency and percentage, and continuous variables as mean ± standard deviation. The optimal cutoff value for the HALP score was determined using X-tile software (Yale University, New Haven) based on OS data, employing the minimum *P* value approach to select the optimal cut point. Differences in categorical variables between HALP groups were assessed using Pearson chi-square test. Sample size consideration: With 18 pCR events and 4 radiomics features + 1 HALP variable, the events-per-variable ratio was 3.6, below the recommended minimum of 10 to 20 events per predictor. Therefore, our results should be considered exploratory and hypothesis-generating; external validation is mandatory before clinical application. Three predictive models were constructed: radiomics-only model model: random forest classifier (ntree = 500, mtry = √*P*) using the 4 selected radiomics features; HALP model: binary HALP score (low ≤ 48.4 vs high > 48.4); combined model: random forest classifier integrating both the 4 radiomic features and the HALP score. For class imbalance (pCR rate 13.1%), the random forest model employed class_weight = “balanced” to assign higher weight to the minority class. Model performance was evaluated using the AUC, sensitivity, specificity, calibration curves, and decision curve analysis. Statistical comparisons between AUC values were performed using DeLong test. For OS analysis, overall survival was defined as the time from NCRT initiation to death from any cause; patients alive at the last follow-up (January 1, 2024) were censored. Multivariate Cox regression was used for OS analysis, with hazard ratios (HRs) and 95% confidence intervals (CIs) reported. Internal validation was conducted via 1000 bootstrap samples to correct for overfitting optimism. Time-to-event outcomes were visualized using Kaplan–Meier curves with log-rank tests. Random forest prognostic plots were generated using R software (version 4.2.3; R Foundation for Statistical Computing, Vienna, Austria, https://www.r‑project.org). All statistical analyses were conducted using SPSS (version 26; IBM Corp., Armonk), R (version 4.2.3), and X-tile (v3.6.1). A 2-tailed *P* value <.05 was considered statistically significant.

## 3. Results

### 3.1. Patient characteristics

Based on the inclusion criteria, 37 of the 187 enrolled patients were excluded, primarily due to incomplete follow-up information (22 cases), distant metastasis (11 cases), and loss to follow-up (4 cases). Among the remaining 150 patients, an additional 13 were excluded – 10 due to treatment complications and 3 who failed to complete treatment for personal reasons. Ultimately, 137 patients were enrolled in the study, including 93 males, with a median age at diagnosis of 58 years (range: 22–81 years). According to the tumor-node-metastasis staging system, 128 patients (93%) had stage III disease and 9 (7%) had stage IVA disease. pCR was achieved in 18 patients, resulting in an overall pCR rate of 13% (Table 1).

**Table 1:** The base patients’ information.

Characteristics		*P*	Characteristics		*P*
Gender		.36	Synchronous chemotherapy		.09
Male	93 (67.8%)		Double drug	128 (93.4%)	
Female	44 (32.2%)		Single drug	9 (6.6%)	
Age (yr)			Differentiation		.45
Range	22–81		Moderate/poor	96 (70.1%)	
Median	58		Well	41 (29.9%)	
ZPS score		.86	Radiotherapy plan		.012
0–1	115 (83.9%)		Complete	116 (84.7%)	
2	22 (16.1%)		Incomplete	21 (15.3%)	
Location		.47			
Upper	45 (32.8%)				
Lower	92 (67.2%)				
Tumor invasion (T-stage)		.17			
T2	5 (3.6%)				
T3	92 (67.2%)				
T4	40 (29.2%)				
Lymph node metastasis (N-stage)		.21			
N1	54 (39.4%)				
N2–3	83 (60.6%)				
cTNM stage		.78			
IIB–Ⅲ	128 (93.4%)				
IVA	9 (6.6%)				

cTNM stage = clinical tumor-node-metastasis stage, N-stage = lymph node metastasis, T-stage = tumor invasion, ZPS score = Zubrod-ECOG WHO performance status.

### 3.2. Radiomics model for pCR prediction

**Figure 2.**
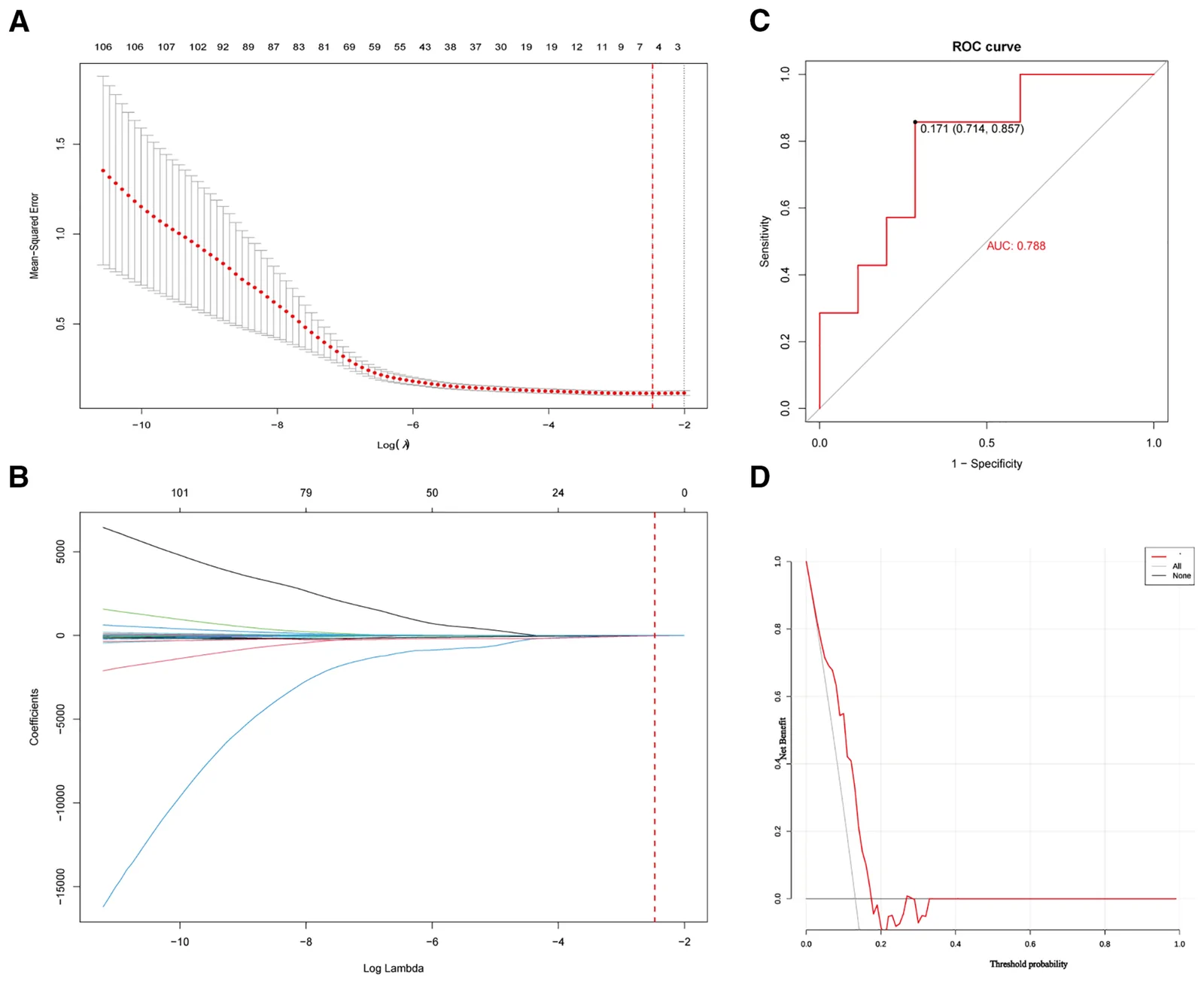
Radiomics model for pCR prediction. (A) LASSO coefficient path. (B) Calibration curve (slope = 0.96). (C) ROC with 95% CI shadow (AUC: 0.788, Delong CI: 0.70–0.87). (D) Decision curve analysis. AUC = area under the curve, CI = confidence interval, LASSO = least absolute shrinkage and selection operator, pCR = pathological complete response, ROC = receiver operating characteristic.

Four selected features remained after least absolute shrinkage and selection operator regression (Fig. 2A and B). The radiomics-only model model achieved an AUC of 0.788 (95% CI: 0.70–0.87), with a sensitivity of 0.714 and a specificity of 0.857. The calibration slope was 0.96 (Fig. 2C). Decision curve analysis showed net benefit at threshold probabilities of 20% to 70% (Fig. 2D). The bootstrap-corrected AUC was 0.77 (1000 iterations), indicating minimal overfitting for the pCR prediction model.

### 3.3. HALP score for evaluating short-term efficacy and long-term prognosis

Based on X-tile analysis using OS as the outcome, the optimal cutoff value of HALP scores was set at 48.4. The enrolled patients were divided into a low HALP group (L-HALP) and a high HALP group (H-HALP), which involved 69 and 68 patients respectively. There were 8 and 10 patients with pCR in the L-HALP and H-HALP groups respectively, showing no significant difference (*P* = .59). As shown in Table 2, the several baseline parameters examined showed no significant differences between the 2 HALP groups (Table 2).

**Table 2:** Differences in basal parameters under different HALP group.

Characteristics	L-HALP (69)	H-HALP (68)	*P*	Characteristics	L-HALP (69)	H-HALP (68)	*P*
Gender			.24	pCR			.59
Male	50 (72.5%)	43 (63.2%)		Yes	61 (88.4%)	58 (85.3%)	
Female	19 (27.5%)	25 (36.8%)		No	8 (11.6%)	10 (14.7%)	
Age (yr)			.42				
≥65	49 (71.0%)	44 (64.7%)					
<65	20 (29%)	24 (35.3%)					
ECOG			.33				
0–1	60 (87%)	55 (80.9%)					
2	9 (13%)	13 (19.1%)					
Location			.627				
Upper	24 (31.8%)	21 (30.9%)					
Lower	45 (69.2%)	47 (69.1%)					
T stage			.28				
T1–2	3 (4.3%)	2 (2.9%)					
T3–4a	66 (95.7%)	66 (97.1%)					
N stage			.18				
N0–1	31 (44.9%)	23 (33.8%)					
N2–3b	38 (55.1%)	45 (66.2%)					
pTNM stage			.78				
IIB–III	63 (91.3%)	65 (95.6%)					
IVA	4 (9.7%)	5 (4.4%)					
Differentiation			.32				
Well	18 (26.1%)	23 (33.8%)					
Moderate/poor	51 (73.9%)	45 (66.2%)					
M stage			.71				
Yes	4 (5.8%)	5 (7.4%)					
No	65 (94.2%)	63 (92.6%)					

cTNM stage = clinical TNM stage, HALP = hemoglobin, albumin, lymphocyte, and platelet score, H-HALP = high HALP, L-HALP = low HALP, N-stage = lymph node metastasis, T-stage = tumor invasion, ZPS score = Zubrod-ECOG WHO performance status.

**Figure 3.**
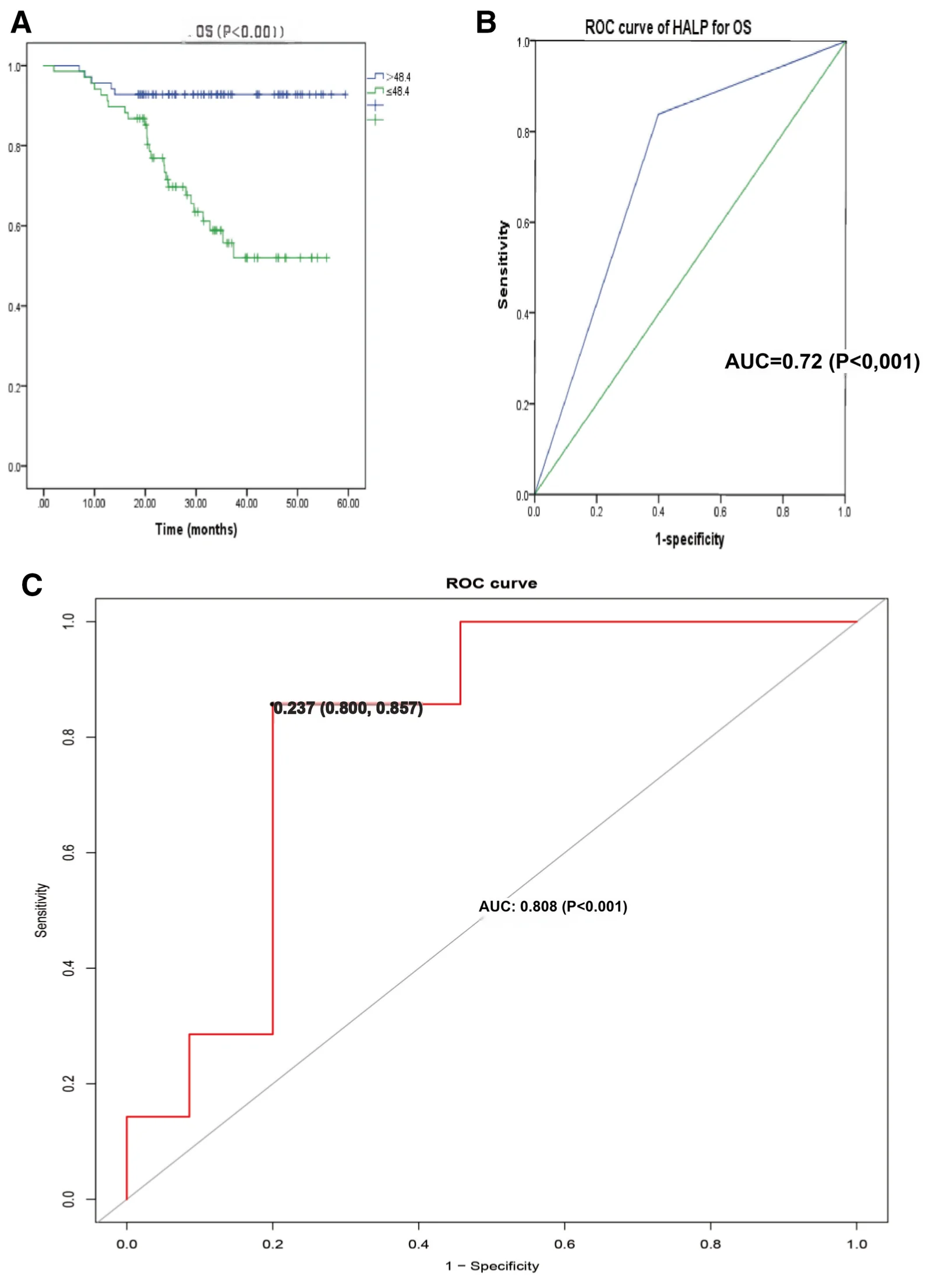
(A) Kaplan–Meier OS curves by HALP score (cutoff 48.4). Shaded areas represent 95% confidence bands; log-rank *P* < .001. B. ROC curve for HALP predicting OS (AUC = 0.72, 95% CI: 0.625–0.818, *P* < .001). C. ROC curve for the combined model predicting pCR (AUC = 0.808, 95% CI: 0.73–0.88, sensitivity: 0.80, specificity: 0.857). AUC = area under the curve, CI = confidence interval, HALP = hemoglobin, albumin, lymphocytes, platelets, OS = overall survival, pCR = pathological complete response, ROC = receiver operating characteristic.

The median follow-up time for all enrolled patients was 29.5 months (range: 2.0–59.4 months). One-way analysis of variance tested the impact of HALP scores on patient OS, showing that lower HALP scores corresponded to worse prognosis (*P* < .001). Moreover, univariate and multivariate Cox regression analyses revealed that HALP was an independent factor for OS in rectal cancer patients undergoing NCRT (HR: 5.83, 95% CI: 2.23–15.2, *P* < .001) (Fig. 3A). At the end of follow-up, receiver operating characteristic curve analysis was performed using death as the independent variable to evaluate the prognostic performance of HALP parameters. The receiver operating characteristic analysis showed that the AUC for OS predicted by HALP was 0.72 (95% CI: 0.625–0.818, *P* < .001) (Fig. 3B).

Sensitivity analysis: When alternative HALP cutoffs reported in the literature (45.6 and 50.2) were applied, the direction of association remained consistent (HR range: 4.92–6.15), suggesting relative robustness of the HALP–OS association to cutoff selection.

### 3.4. Combined model for pCR prediction

The AUC of the combined model was 0.808 (sensitivity: 0.80, specificity: 0.857, *P* < .001), demonstrating better predictive value than the pure radiomics model (Fig. 3C).

### 3.5. Combined model for overall survival prediction

**Figure 4.**
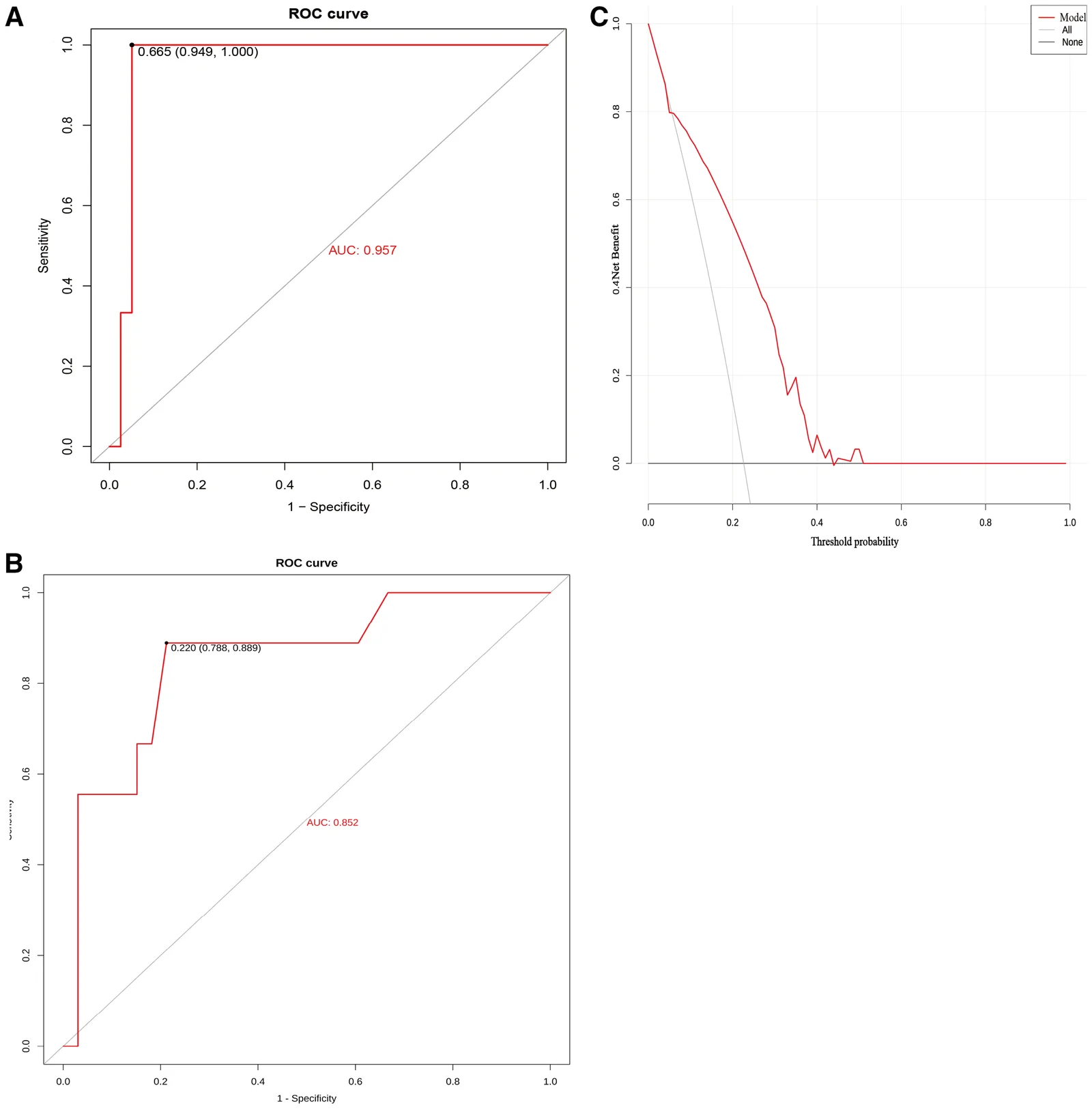
Combined model for OS prediction. (A) ROC (apparent AUC: 0.957). (B) The corrected AUC: 0.85. (C) DCA. AUC = area under the curve, DCA = decision curve analysis, OS = overall survival, ROC = receiver operating characteristic

The performance metrics showed an apparent AUC of 0.957, with a sensitivity of 0.945 and a specificity of 1.0. The bootstrap-corrected AUC was 0.852 (95% CI: 0.788–0.889), which remained significantly higher than the HALP-alone model (0.72) (DeLong *P* = .002). Decision curve analysis demonstrated net benefit at threshold probabilities of 10% to 60% (Fig. 4A–C). Kaplan–Meier analysis confirmed that patients in the high-risk group (as defined by the combined model) had significantly worse OS compared to the low-risk group (log-rank *P* < .001).

## 4. Discussion

The combined HALP score is a promising indicator of nutritional and inflammatory status, and its prognostic value has been demonstrated in various solid tumors.^[22]^ The present study shows that lower HALP is associated with worse prognosis, consistent with the findings of Yordanagil et al.^[23]^ Moreover, HALP is an independent prognostic factor for LARC patients (HR: 5.83, 95% CI: 2.23–15.2, *P* < .001), which is also consistent with some previous studies.^[16,24]^ Additionally, the cutoff value of HALP determined by X-tile analysis was 48.4. Enrolled patients were divided into 2 groups based on this cutoff. Single-factor analysis of variance found no significant differences in baseline parameters or pCR rates between the 2 groups. This result is inconsistent with the reported significant correlation between HALP components and N stage,^[25]^ which may be because HALP does not have a standardized cutoff value, or there may be other biological factors. We will conduct further external verification to summarize this result.

Radiomics plays an important role in treatment guidance, preoperative evaluation, and timely detection of distant metastasis for rectal cancer.^[26]^ MRI has become the most appropriate method to evaluate the local status of rectal cancer and plays an important role in primary staging, selection of LARC patients suitable for NCRT, and identification of adverse prognostic factors.

We developed and internally validated a T2W MRI radiomics and HALP fusion model that achieved excellent discrimination for both pCR (AUC: 0.81) and OS (AUC: 0.85 after correction) in LARC patients undergoing NCRT. We compared this with representative recent radiomics studies in rectal cancer (2019–2024). The median AUC of external literature was 0.81 (range: 0.76–0.85). Our corrected AUC (0.85) ranks in the top 25%, despite our smaller sample size. Key differences include: most studies used diffusion-weighted imaging or dynamic contrast‑enhanced MRI, while we used T2W only, which is easier to generalize. Only 2 studies integrated clinical variables, and none used HALP (Table 3). To our knowledge, this is the 1st study to integrate systemic immunity-nutrition status (HALP) with tumor heterogeneity metrics derived from pretreatment T2W MRI, providing a cost-neutral decision support tool. Biological plausibility is supported by the fact that radiomics features (low-gray-level run emphasis, etc) mirror intra-tumor hypoxia and fibrosis, known predictors of poor response. HALP captures host inflammatory reserve; low HALP reflects anemia, malnutrition, and immunosuppression, all linked to impaired DNA damage repair and worse NCRT efficacy. Our corrected AUC (0.85) ranks within the top quartile of recent multicenter studies (Shin et al^[12]^ [2022]: 0.82; Shaish et al^[13]^ [2020]: 0.81) while requiring only T2W sequences and routine blood counts, facilitating widespread adoption.

**Table 3:** Head-to-head comparison of literature (extended).

Study (year)	MRI sequences	Algorithm	Patients (n)	EV*	AUC
Shin et al^[12]^ (2022)	T2W + DWI	RF	521	Yes	0.82
Shaish et al^[13]^ (2020)	T2W + DWI	XGBoost	802	Yes	0.81
Horvat et al^[27]^ (2018)	T2W	CNN	318	No	0.79

AUC = area under the curve, CNN = convolutional neural network, DWI = diffusion-weighted imaging, EV = external validation, RF = random forest, T2W = T2-weighted.

* Bootstrap-corrected.

However, this study has several limitations: First, single-center, retrospective design; selection bias cannot be excluded. Second, low pCR rate (13.1%), possibly reflecting strict pathological review; multicenter validation is essential in the future. Third, limited sample size and events-per-variable ratio: With only 18 pCR events and 5 predictors, the events-per-variable ratio was 3.6, well below the recommended minimum of 10 to 20.^[28]^ This substantially increases the risk of overfitting and limits the generalizability of our findings. The substantial AUC shrinkage from 0.957 to 0.852 in the OS model (a drop of 0.105) is a direct manifestation of this overfitting risk. Fourth, cutoff optimization bias: The HALP cutoff of 48.4 was derived from the same dataset used for model development, which may introduce optimism bias. Although sensitivity analyses using alternative cutoffs yielded consistent directional findings, this does not replace independent validation. Fifth, absence of external validation; internal validation showed AUC shrinkage from 0.96 to 0.85, indicating potential overfitting. Sixth, lack of test-retest or cross-scanner reproducibility assessment for radiomics features. Seventh, lack of molecular data: TP53, KRAS, BRAF, and MSI status were unavailable; future work will integrate genomic mutations and microsatellite instability.

Future directions: A prospective multicenter trial will enroll additional patients to perform external validation. Integration of molecular biomarkers: TP53, KRAS, BRAF, SMAD4, and PTEN mutations and microsatellite instability will be incorporated into a multi-omics nomogram. Advanced MRI sequences: diffusion-weighted imaging (apparent diffusion coefficient histogram) and dynamic contrast-enhanced (Ktrans, Ve) will be added to improve micro-environmental characterization. Dosimetric parameters: dose-volume histogram metrics (bowel V45, bladder V50) and radiobiological equivalent dose will be tested for synergistic value. Blood-based biomarkers: circulating interleukin-6, C-reactive protein, neutrophil extracellular traps, and fecal microbiome diversity will be explored as dynamic predictors. A federated learning framework will be adopted to preserve patient privacy while leveraging cross-institutional data.

## 5. Conclusions

In this retrospective single-center study, a fusion model combining T2W MRI radiomics and HALP score achieved good discrimination for both pathological complete response and overall survival in LARC patients undergoing NCRT. However, the apparently high AUC (0.957) was downgraded to 0.85 after correction for optimism, and external validation is mandatory before any clinical deployment. Given the limited sample size and low events-per-variable ratio, these findings should be considered exploratory and hypothesis-generating. Prospective multicenter trials incorporating molecular and dosimetric data are underway to confirm these preliminary findings.

## Acknowledgments

We thank the patients and their families for participating in this study. We acknowledge the radiology and pathology staff of Jurong Hospital and the First Affiliated Hospital of Nanjing Medical University for data collection.

## Author contributions

**Conceptualization:** Xiaojiao Chen, Sheng Zhang, Xinchen Sun, Ning Xue.

**Data curation:** Xiaolei Wan, Xiaojiao Chen, Caiqiang Zhu, Dan Mei, Sheng Zhang, Xiaolin Ge, Xinchen Sun, Zhengqi Li, Xiaoke Di.

**Formal analysis:** Xiaolei Wan, Xiaojiao Chen, Caiqiang Zhu, Dan Mei, Xiaolin Ge, Zhengqi Li, Xiaoke Di.

**Investigation:** Xiaolin Ge, Xiaoke Di, Ning Xue.

**Methodology:** Xiaolei Wan, Caiqiang Zhu, Dan Mei, Xiaolin Ge, Xinchen Sun, Xiaoke Di, Ning Xue.

**Project administration:** Xiaolei Wan, Xiaojiao Chen, Xiaolin Ge.

**Resources:** Xiaolei Wan, Liang Liang.

**Software:** Xiaolei Wan, Zhengqi Li, Ning Xue.

**Supervision:** Xiaojiao Chen, Liang Liang, Xiaolin Ge, Xinchen Sun, Ning Xue.

**Validation:** Xiaojiao Chen, Liang Liang, Sheng Zhang, Xinchen Sun, Ning Xue.

**Visualization:** Xiaojiao Chen, Liang Liang, Sheng Zhang, Xinchen Sun, Ning Xue.

**Writing – original draft:** Xiaolei Wan.

**Writing – review & editing:** Xiaolei Wan.



## References

[1] Siegel RL Miller KD Fedewa SA. Colorectal cancer statistics, 2017. CA Cancer J Clin. 2017;67:177–93. 28248415 10.3322/caac.21395

[2] Glynne-Jones R Wyrwicz L Tiret E. Rectal cancer: ESMO clinical practice guidelines for diagnosis, treatment and follow-up. Annal Oncol. 2017;28(suppl_4):iv22–iv40. 10.1093/annonc/mdx224 28881920

[3] Maas M Nelemans PJ Valentini V. Long-term outcome in patients with a pathological complete response after chemoradiation for rectal cancer: a pooled analysis of individual patient data. Lancet Oncol. 2010;11:835–44. 20692872 10.1016/S1470-2045(10)70172-8

[4] Ludmir EB Palta M Willett CG Czito BG. Total neoadjuvant therapy for rectal cancer: an emerging option. Cancer. 2017;123:1497–506. 28295220 10.1002/cncr.30600

[5] Giannini V Mazzetti S Bertotto I. Predicting locally advanced rectal cancer response to neoadjuvant therapy with 18F-FDG PET and MRI radiomics features. Eur J Nucl Med Mol Imaging. 2019;46:878–88. 30637502 10.1007/s00259-018-4250-6

[6] Siegel RL Miller KD Jemal A. Cancer statistics, 2018. CA Cancer J Clin. 2018;68:7–30. 29313949 10.3322/caac.21442

[7] Ferlay J Colombet M Soerjomataram I. Estimating the global cancer incidence and mortality in 2018: GLOBOCAN sources and methods. Int J Cancer. 2019;144:1941–53. 30350310 10.1002/ijc.31937

[8] Benson AB Venook AP Al-Hawary MM. Rectal cancer, version 2.2018, NCCN clinical practice guidelines in oncology. J Nat Comprehensive Cancer Network. 2018;16:874–901. 10.6004/jnccn.2018.0061 PMC10203817 30006429

[9] Martens MH Maas M Heijnen LA. Long-term outcome of an organ preservation program after neoadjuvant treatment for rectal cancer. J Natl Cancer Inst. 2016;108:djw171. 27509881 10.1093/jnci/djw171

[10] Cui Y Yang X Shi Z. Radiomics analysis of multiparametric MRI for prediction of pathological complete response to neoadjuvant chemoradiotherapy in locally advanced rectal cancer. Eur Radiol. 2019;29:1211–20. 30128616 10.1007/s00330-018-5683-9

[11] Sun Y Hu P Wang J. Radiomic features of pretreatment MRI could identify T stage in patients with rectal cancer: preliminary findings [published online ahead of print February 13, 2018]. J Magn Reson Imaging. doi: 10.1002/jmri.25969. 10.1002/jmri.25969 29437279

[12] Shin J Seo N Baek SE. MRI radiomics model predicts pathologic complete response of rectal cancer following chemoradiotherapy. Radiology. 2022;303:351–8. 35133200 10.1148/radiol.211986

[13] Shaish H Aukerman A Vanguri R. Radiomics of MRI for pretreatment prediction of pathologic complete response, tumor regression grade, and neoadjuvant rectal score in patients with locally advanced rectal cancer undergoing neoadjuvant chemoradiation: an international multicenter study. Eur Radiol. 2020;30:6263–73. 32500192 10.1007/s00330-020-06968-6

[14] Diakos CI Charles KA McMillan DC Clarke SJ. Cancer-related inflammation and treatment effectiveness. Lancet Oncol. 2014;15:e493–503. 25281468 10.1016/S1470-2045(14)70263-3

[15] Jiang H Li H Li A. Preoperative combined hemoglobin, albumin, lymphocyte and platelet levels predict survival in patients with locally advanced colorectal cancer. Oncotarget. 2016;7:72076–83. 27765916 10.18632/oncotarget.12271 PMC5342146

[16] Chen XL Xue L Wang W. Prognostic significance of the combination of preoperative hemoglobin, albumin, lymphocyte and platelet in patients with gastric carcinoma: a retrospective cohort study. Oncotarget. 2015;6:41370–82. 26497995 10.18632/oncotarget.5629 PMC4747412

[17] Zhang X Kiapour N Kapoor S. IL-11 induces encephalitogenic Th17 cells in multiple sclerosis and experimental autoimmune encephalomyelitis. J Immunol. 2019;203:1142–50. 31341075 10.4049/jimmunol.1900311 PMC6697738

[18] Tarique M Naz H Suhail M. Differential expression of programmed death 1 (PD-1) on various immune cells and its role in human leprosy. Front Immunol. 2023;14:1138145. 37153623 10.3389/fimmu.2023.1138145 PMC10161389

[19] Seyedsadr M Wang Y Elzoheiry M. IL-11 induces NLRP3 inflammasome activation in monocytes and inflammatory cell migration to the central nervous system. Proc Natl Acad Sci USA. 2023;120:e2221007120. 37339207 10.1073/pnas.2221007120 PMC10293805

[20] Khan TH Srivastava N Srivastava A. SHP-1 plays a crucial role in CD40 signaling reciprocity. J Immunol. 2014;193:3644–53. 25187664 10.4049/jimmunol.1400620

[21] Peng D Zhang CJ Tang Q. Prognostic significance of the combination of preoperative hemoglobin and albumin levels and lymphocyte and platelet counts (HALP) in patients with renal cell carcinoma after nephrectomy. BMC Urol. 2018;18:20. 29544476 10.1186/s12894-018-0333-8 PMC5855974

[22] Wang X He Q Liang H. A novel robust nomogram based on preoperative hemoglobin and albumin levels and lymphocyte and platelet counts (HALP) for predicting lymph node metastasis of gastric cancer. J Gastrointestinal Oncol. 2021;12:2706–18. 10.21037/jgo-21-507 PMC8748024 35070400

[23] Yordanagil M Bakir H Güler Avci G Yildirim M Ozkan N Ismail O. Do haematological parameters such as HALP and Lymphocyte to C-reactive protein ratio predict tumor response to neoadjuvant chemoradiotherapy in locally advanced rectal cancer? Polski Przeglad Chirurgiczny. 2022;95:1–5. 10.5604/01.3001.0016.0959 36805993

[24] Guo Y Shi D Zhang J. The hemoglobin, albumin, lymphocyte, and platelet (HALP) score is a novel significant prognostic factor for patients with metastatic prostate cancer undergoing cytoreductive radical prostatectomy. J Cancer. 2019;10:81–91. 30662528 10.7150/jca.27210 PMC6329846

[25] Zhang D Chen S Cao W Geng N Feng C. HALP score based on hemoglobin, albumin, lymphocyte and platelet can predict the prognosis of tongue squamous cell carcinoma patients. Heliyon. 2023;9:e20126. 37809958 10.1016/j.heliyon.2023.e20126 PMC10559844

[26] Taylor SA Mallett S Beare S. Diagnostic accuracy of whole-body MRI versus standard imaging pathways for metastatic disease in newly diagnosed colorectal cancer: the prospective Streamline C trial. Lancet Gastroenterol Hepatol. 2019;4:529–37. 31080095 10.1016/S2468-1253(19)30056-1 PMC6547166

[27] Horvat N Veeraraghavan H Khan M. MR imaging of rectal cancer: radiomics analysis to assess treatment response after neoadjuvant therapy. Radiology. 2018;287:833–43. 29514017 10.1148/radiol.2018172300 PMC5978457

[28] Peduzzi P Concato J Kemper E Holford TR Feinstein AR. A simulation study of the number of events per variable in logistic regression analysis. J Clin Epidemiol. 1996;49:1373–9. 8970487 10.1016/s0895-4356(96)00236-3

