# Supplementary material for: Efficacy of HALP combined with T2-weighted MRI radiomics in predicting pathological complete response and overall survival in rectal cancer after neoadjuvant chemoradiotherapy: A retrospective single-center study in China
Source: Medicine (Baltimore). 2026 Sep 11;105(37):e50554. doi: 10.1097/MD.0000000000050554 (PMC13574486; doi:10.1097/MD.0000000000050554)
Supplement: Supplementary file 1 [file medi-105-e50554-s001.docx]

Table S1 Split consistency ICC

| **Feature category** | Reader 1 vs 2 | ICC (95 % CI) | Interpretation |
| --- | --- | --- | --- |
| **First-order statistics** | 0.89 | 0.82-0.94 | Excellent |
| **GLCM** | 0.91 | 0.85-0.95 | Excellent |
| **GLRLM** | 0.88 | 0.80-0.93 | Good |
| **Shape features** | 0.94 | 0.90-0.97 | Excellent |
| **LoG (σ=1 mm)** | 0.86 | 0.78-0.92 | Good |

GLCM：gray-level co-occurrence matrix ;GLRLM; gray-level run-length matrix , GLSZM:gray-level size zone matrix;LoG: Laplacian of Gaussian features;
